# In Vitro and In Vivo Antidiabetic Activity, Phenolic Content and Microscopical Characterization of *Terfezia claveryi*

**DOI:** 10.3390/molecules27154843

**Published:** 2022-07-28

**Authors:** Ala’ Abu-Odeh, Mayadah Shehadeh, Ghadeer A. R. Y. Suaifan, Nida Karameh, Diana Abdel Rahman, Yasser Kandil

**Affiliations:** 1 Department of Pharmaceutical Chemistry and Pharmacognosy, Faculty of Pharmacy, Applied Science Private University, Amman 11931, Jordan; a_abuodeh@asu.edu.jo; 2 Department of Pharmaceutical Sciences, School of Pharmacy, The University of Jordan, Amman 11942, Jordan; gh.suaifan@ju.edu.jo (G.A.R.Y.S.); diana.rehmen@gmail.com (D.A.R.); 3 Pharmacological and Diagnostic Research Center (PDRC), Faculty of Pharmacy, Al-Ahliyya Amman University, Amman 19328, Jordan; n.karameh@ammanu.edu.jo; 4 Department of Biochemistry and Molecular Biology, Faculty of Pharmacy, Al-Azhar University, Cairo 4434004, Egypt; kandil.yasser@azhar.edu.eg

**Keywords:** diabetes, truffle, *Terfezia claveryi*, total phenols, α-glucosidase inhibition, antihyperglycemic, high-fat diet, alloxan, type 2 diabetes mice model

## Abstract

*Terfezia claveryi* (*T. claveryi*) is used by traditional healers in the Middle East region to treat several diseases, including diabetes. The present study evaluated the total phenolic and investigated the blood-glucose-lowering potential of different aqueous extracts of this selected truffle using in vitro and in vivo models. The phytochemical profile was examined using UPLC-MS. The macerate and the microwave-assisted extract were the richest in phenolic compounds. All *T. claveryi* extracts exhibited a remarkable α-glucosidase inhibitory effect in vitro, with an IC_50_ of 2.43, 3.26, 5.18 and 3.31 mg/mL for the aqueous microwave-assisted extract macerate, infusion and decoction, respectively. On the other hand, in the high-fat diet alloxan-induced diabetic mice model, all tested crude aqueous extracts exhibited a significant antihyperglycemic activity (*p* < 0.05). Four hours after the administration of the 250 mg/kg dose, the macerate was able to induce a 29.4% blood-glucose-lowering effect compared to a 24.8% reduction induced by the infusion, which was sustained for a further two hours. The hypoglycemic effect (29.3% and 32.4%) was also recorded six hours after the administration of the single dose 500 mg/kg of the macerate and the infusion, respectively. Truffle extracts exhibited antidiabetic activity both in vitro and in vivo, providing a rationale for the traditional use as a natural hypoglycemic.

## 1. Introduction

Diabetes mellitus is a chronic progressive multifactorial metabolic disorder characterized by carbohydrates, lipids and protein metabolism disturbances. The hallmark of diabetes is sustained hyperglycemia [1]. It occurs due to the total loss of function of β cells of islets of Langerhans (type 1) or due to insulin deficiency and resistance (type 2) [2]. Diabetes is considered an epidemic disease. The WHO expects the number of diabetic patients to reach 300 million by the end of 2025 [3].

There are numerous approaches and medications to treat diabetes. The current antidiabetic medications, including insulin and agents, have several limitations, including long-term treatment, adverse side effects and high cost. These limitations have increased the tendency to search for effective and affordable medications with fewer side effects [4]. However, complementary and alternative medicine interventions, including herbal and nutritional interventions in patients with diabetes, are being used increasingly to counter the disease [5]. 

Plants have always played an essential role in traditional medicine to treat various diseases. They contain several metabolites that have complementary or synergistic effects on the human body. They rarely cause adverse effects if recommended by qualified healthcare personnel [6].

*Terfezia claveryi* (Pezizaceae) is an edible macrohypogeous fungus used in many cultures due to its medicinal and nutritional properties. It is distributed in countries around the Mediterranean region and the Arabian Peninsula. Some desert *T. claveryi* were also found in South Africa and Botswana [7,8,9,10]. The most common name of desert *T. claveryi* in the Arabian region is Kamah [11].

*Terfezia claveryi* is a source of carbohydrates, amino acids (methionine, cysteine, tryptophan and lysine), proteins, fats, fibers, minerals and ascorbic acids [8,12]. In addition, it contains phenolic compounds and tocopherols in minor amounts [10,13]. Mushrooms, including *Terfezia claveryi*, are considered an extensive source of bioactive peptides. Consequently, their composition and secondary structure display various biological activities [14].

*Terfezia* species have been reported to be rich in proteins and are usually recommended as meat substitute in arid and semiarid areas of the Mediterranean region. Prophet Mohammed mentioned the Kama for its beneficial effect in treating eye infections. Later, Ibn- Sina described it as healing for vomiting, wounds and weakness [9]. It is also used to treat skin diseases, wounds and as an aphrodisiac [7]. Several biological activities have been explored for *Terfezia claveryi,* including antibacterial, antioxidant, potential hepatoprotective, antimutagenic, anti-inflammatory and anticancer activities [12]. The antioxidant property of truffles has elevated the question of whether they can be used as a functional/medical food that could play a role in health maintenance and disease prevention, including diabetes [15].

Recently, the methanol extract of *T. claveryi* was reported to be a stronger inhibitor of the α-amylase enzyme than acarbose in vitro. In addition, the extract reduced the fasting blood glucose levels in diabetic rats when tested in vivo [16].

The present study was designed to evaluate the in vitro and in vivo antidiabetic activity of the *Terfezia claveryi* aqueous crude extracts using different extraction techniques, to identify the microscopical fingerprinting and to quantify the phenolic content in each prepared extract. To our knowledge, it is the first study to investigate the antidiabetic activity of the aqueous extract of *T. claveryi* obtained by a different method of extraction.

## 2. Results

### 2.1. Microscopical Examination

**Figure 1:**
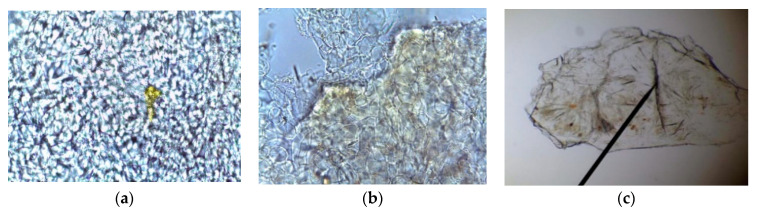
Microscopic characterization of *Terfezia claveryi*. (**a**) Epidermis cells; (**b**) Oil glands; (**c**) Sclereid-like structure in the peridium.

Microscopy is used as part of the identity authentication, taxonomy, plant tissue anatomy and quality control of plant material [17]. The epidermal cells possessed thin walls with oil glands concentrated near each other. In addition, the truffle tuber peridium (outer skin) showed a unique sclereid-like structure (Figure 1).

### 2.2. Preliminary Phytochemical Screening

Preliminary qualitative analysis of *T. claveryi* aqueous extracts for the bioactive phytochemical constituents indicated the presence of carbohydrates, flavonoids, proteins, saponins and terpenes. No alkaloids were detected in any of the tested extracts.

### 2.3. Determination of Total Phenolic Content of the Crude Extracts

The Folin–Ciocalteu colorimetric method was used to determine the total phenolic content of the extracts. The macerate and the microwave-assisted extracts were found to be the richest in phenolic compounds at (18.5 ± 0.55 mg GAE/g) and (16.9 ± 0.09 GAE/g), respectively, compared to extracts obtained by the decoction and the infusion methods, where the content was (6.8 ± 1.8 GAE/g) and (5.0 ± 0.10 GAE/g), respectively.

### 2.4. UPLC-ESI-MS

**Figure 2:**
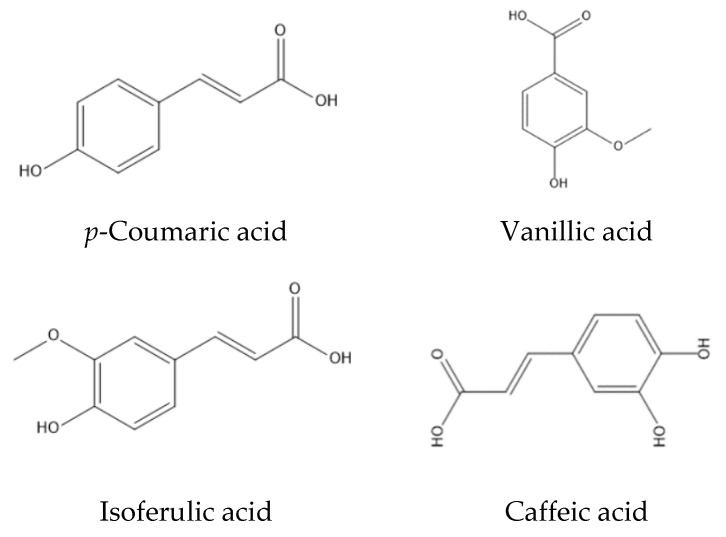
Chemical structures of the phenolic acids found in *Terfezia claveryi*.

**Figure 3:**
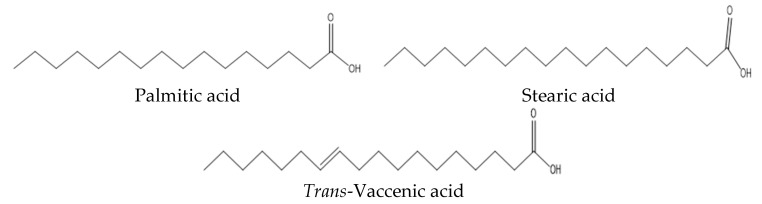
Chemical structures of the fatty acids found in *Terfezia claveryi*.

In all *T. claveryi* extracts, caffeic acid, coumaric acid, protocatechuic aldehyde, ***trans***-vaccenic acid and vanillic acid appeared as molecular ions of M-H- at *m/z* (179.03501, 163.04002, 137.02452, 281.24855 and 167.03451), respectively (Table 1). The 3, 5-dimethoxy-4-hydroxy acetophenone had a molecular ion at *m/z* (195.0466) and a fragment at *m/z* (133.0108), indicating a loss of two methoxy groups. Palmitic and stearic acids were detected at *m/z* (255.2325 and 284.27123), respectively, with a fragment at *m/z* (44.9974), corresponding to the loss of a COOH from the precursor [M − H]–ion, see (Figure 2 and Figure 3).

Scopoletin was detected at *m/z* (191.0347), with a major fragment at *m/z* (44.9722), indicating a loss of (COOH). In addition, 3, 5-dimethoxy-4-hydroxy acetophenone was detected at *m/z* 195.0669. Isoferulic acid was detected at *m/z* (193.051), with a major fragment at *m/z* (44.9988), representing COOH. Carbohydrates are the main macronutrients present in truffles. In all the analyzed truffle extracts, piscopyranose, mannitol and trehalose peaks were detected between 0.59 and 1.26 min.

### 2.5. In Vitro α-Glucosidase Inhibition Activity of Terfezia claveryi Extracts

**Figure 4:**
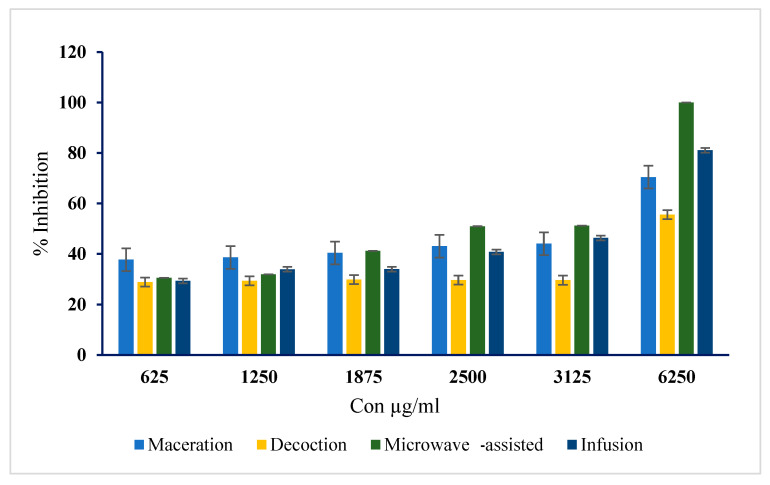
Inhibition of α-glucosidase activity by different *T. claveryi* aqueous extracts.

The in vitro α-glucosidase inhibition potential of the *T. claveryi* extracts was evaluated. The percentage of the enzyme inhibition as a function of the concentration and the type was detected. The IC_50_ was calculated from the graph (Figure 4). Concentration-dependent inhibitory activity was observed for all tested extracts, as well as the standard at various concentrations.

The microwave-assisted extract exhibited the lowest IC_50_ of 2.43 ± 0.02 mg/mL. The IC_50_ values of the extracts obtained by infusion, maceration and decoction were 3.26 ± 0.99 mg/mL, 3.31 ± 5.83 mg/mL and 5.18 ± 1.04 mg/mL, respectively. The standard control acarbose exhibited an IC_50_ of 0.33 mg/mL (Table 2).

### 2.6. Antihyperglycemic Activity of Crude Truffle Extracts on High-Fat Diet Alloxan-Induced Diabetic Mice

**Figure 5:**
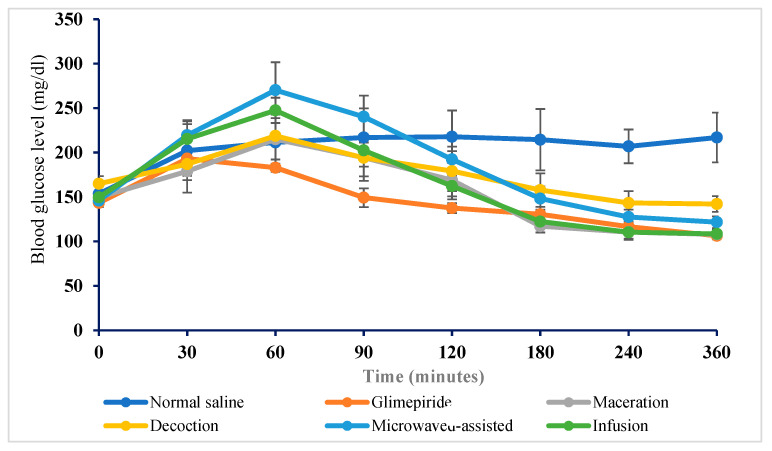
The change in blood glucose level in diabetic mice induced by a single dose of 250 mg/kg *Terfezia claveryi* extracts and glimepiride. Each bar represents the mean ± SEM for (*n* = 6) mice per treated group.

**Figure 6:**
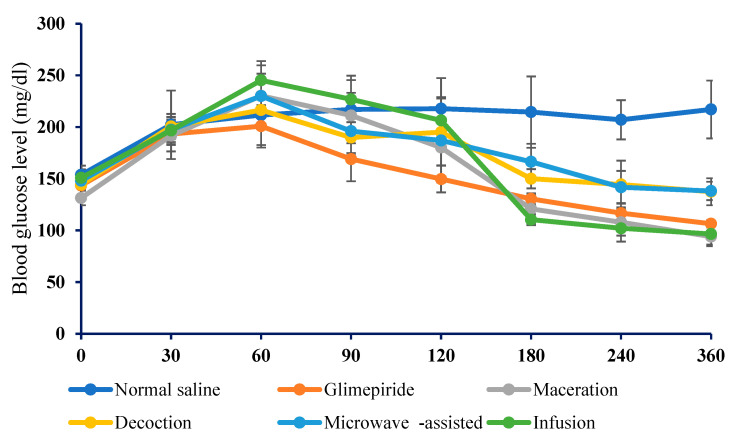
The change in blood glucose level in diabetic mice induced by a single dose of 500 mg/kg *Terfezia claveryi* extracts. Each bar represents the mean ± SEM for (*n* = 6) mice per treated group.

The antihyperglycemic effect of *Terfezia claveryi* extracts on high-fat diet alloxan-induced diabetes is shown in Table 2. Between and within-group analyses were performed to reveal the differences across the various groups and time points between the BGL (blood glucose level). The between-group analysis revealed no significant difference in the baseline fasting BGL across all groups before administering any treatments. However, 60 min later, the blood glucose levels increased when the mice had unrestricted access to water and food compared to baseline fasting BGL, regardless of the treatment (Figure 5 and Figure 6).

Considering the within-group comparison, a BGL reduction effect was observed for all tested doses of the crude extracts at different time points. At both doses of 250 and 500 mg/kg, body weight showed only a weak reduction after 2 h of administration, while glimepiride showed more pronounced antidiabetic activity in the high-fat diet alloxan-induced diabetic mice. At 4 and 6 h, the magnitude of the BGL reduction induced by the macerate and infusion was comparable to that for glimepiride, as shown in Table 3. However, the administration of a dose of 500 mg/kg did not show a higher BGL reduction effect. 

At a dose of 250 mg/kg, the maximum BGL reduction of 26.6%, was recorded for the macerate compared to 8.7%, 17.3 % and 27.8 % reduction induced by the decoction, microwaved-assisted extract and infusion, respectively. On the other hand, the maximum BGL lowering effect of 29.3% was induced in mice that received a dose of 500 mg/kg of the macerate. Other extracts were able to decrease the BGL by 2.7% decoction, 6.0% microwave-assisted and 32.4% the infusion when tested at the same dose.

## 3. Discussion

Extraction is the core step in discovering and evaluating bioactive compounds from plants. Plant material extracts are widely produced by conventional methods, including maceration, decoction, percolation, infusion and hot continuous (reflux and Soxhlet) extraction. However, new methods such as ultrasound-assisted solvent extraction (UASE), microwave-assisted solvent extraction (MASE) and supercritical fluid extractions (SFE) have achieved accumulative interest during the latest decades because they have increased the efficiency of the extraction and are considered eco-friendly techniques [18]. The current study used water as an extraction solvent and different extraction processes were implemented to simulate the traditional preparation method used by herbal healers and the public. Phenolics are the chief antioxidant compounds found in plants. Various chromatographic and spectrophotometric techniques can estimate the plant’s phenolic content. However, the former is expensive, time-consuming and requires many preparation steps. On the other hand, the Folin–Ciocalteu colorimetric assay is an easy, economical and widely used method [19]. It is worth mentioning that the Folin assay is considered an indirect way to measure the antioxidant capacity because it is an oxidation/reduction reaction [20].

Wahiba and coworkers have reported the total phenolic content of the *Terfezia claveryi* methanolic extract at 15 mg/g dry weight gallic acid equivalent [18]. In our study, the highest phenolic content was detected in the macerate (18.5 ± 0.55 mg GAE/g extract), followed by the microwave-assisted (16.9 ± 0.09 GAE/g extract). The decoction and the infusion phenolic content were found to be (6.8 ± 1.8 GAE/g extract) and (5.0 ± 0.10 GAE/g extract), respectively [21]. These findings support the reported effect of the heat-based extraction (infusion and decoction) on the total phenolic content. Generally, the total phenolic content is affected by the sample’s type, chemical and physical properties, solvent’s polarity, extraction method, extraction temperature and extraction time, sample-to-solvent ratio and matrix properties, including the particle size [22].

Throughout the development of type 2 diabetes, cells induce resistance against insulin, decreasing their ability to uptake glucose from the blood. Therefore, the ultimate goal of diabetes treatment is to regain an optimal level of blood glucose, especially after a meal. Postprandial hyperglycemia is the initial stage of the metabolic abnormality that occurs in type 2 diabetes. It contributes to the progression of diabetes and the development of the micro- and macrovascular complications associated with it. Thus, early recognition and targeting contribute to the successful management of diabetes. Inhibitors α-glucosidase, a small intestinal enzyme that catalyzes the final step of carbohydrate digestion of the corresponding disaccharides and monosaccharides, and α-amylase, an enzyme found in saliva and pancreatic juice that breaks down long-chain carbohydrates, are both effective in delaying glucose absorption and managing postprandial hyperglycemia [22,23,24].

In vitro testing is helpful for the mechanistic-based screening of plant extracts and isolated natural products and is recommended as the initial step toward screening potential antidiabetics [25]. Acarbose is widely used in treating patients with type 2 diabetes by hindering the upper gastrointestinal glucosidases in a dose-dependent manner, resulting in delayed glucose absorption and alleviating postprandial hyperglycemia. However, abdominal side effects, such as flatulence and diarrhea or abdominal discomfort, have frequently been reported due to the bacterial fermentation of undigested carbohydrates [26,27].

The α-glucosidase enzyme inhibitory activity is correlated to the concentration of phenolics in the tested extract, as represented by the IC_50_ values (Table 2). The microwave-assisted extract exhibited total inhibition of the enzyme and the lowest IC_50_ value (2.43 mg/mL), which is consistent with the high phenolic content compared to other extracts. On other hand, the inhibition potential of the infusion was found to be high (IC_50_ 3.26 mg/mL) despite being poor in phenolic content, which could be attributed to the presence of other potent bioactive phytochemicals yet to be identified by further research. However, the inhibition of α-glucosidase enzyme activity remains one of the suggested potential mechanisms of action exhibited by truffle extracts [28].

A systemic review summarized 411 natural products isolated from medicinal plants worldwide that exhibited α-glucosidase inhibitory activity. These natural product inhibitors structurally combine alkaloid, terpene, quinine, flavonoid, phenol, phenylpropanoid and steroid skeletons rich in organic acid, alcohol, ester and allyl functional groups [28]. The preliminary phytochemical screening of the macerated crude extract revealed the presence of flavonoids, saponins and terpenes, but there is an absence of alkaloids.

In the current study, the in vitro findings were supported by the in vivo testing results. Animal models for diseases are widely used experimentally to understand the pathophysiology of an illness and help in the drug development process. Through the years, various animal models were developed to study the antidiabetic effects of compounds, including genetic, surgical, chemical and diet-induced models [29].

Mice and rats are commonly used in diabetic research because they share signaling and metabolic pathways with humans and can develop insulin resistance and hyperglycemia, resembling humans. The physiology of mouse/rat models is well documented and comprehended [25].

Mice nourished with a high-fat diet have been reported to induce obesity and type 2 diabetes characteristic features. Additionally, the combination of high-fat feeding with the injection of a chemical agent such as alloxan to induce metabolic disorganization characterizing type 2 diabetes had also been formerly reported [30,31]. Therefore, to confirm the type II model, mice were investigated for insulin resistance using the (HOMA-IR) index. It is well accepted that insulin resistance is a significant feature of obesity–diabetes [31].

The serum glucose level was measured at baseline and there were no significant differences across the groups observed. The normal saline-treated animals did not show a reduction in blood glucose compared to the baseline level. However, significant blood glucose reduction was noticed in diabetic mice after the single dose administration of different *Terfezia claveryi* extracts, indicating that the change in the blood glucose level was attributed to the treatment received. However, the antihyperglycemic effect of the extracts obtained by decoction and microwave-assisted methods was less intensive and immediate than that of glimepiride, suggesting that these extracts might reduce the side effects of hypoglycemia during the treatment of diabetes. In addition, the antihyperglycemic effect of the extracts obtained by the maceration and infusion methods was comparable to glimepiride, suggesting the presence of potent bioactive phytochemicals in the tested extracts to be identified in further studies.

The hypoglycemic activity observed in the glimepiride-treated group was due to the effect induced by the sulfonylurea drug that can selectively block the ATP-sensitive K+ channels (KATP) in the plasma membrane of the pancreatic β-cells, thereby potentiating insulin release. This suggests that the low multiple doses of alloxan intraperitoneal injection did not destroy the β-cells [32]. Thus, the hypoglycemic activity of *Terfezia claveryi* extracts may be due to the potentiation of insulin secretion from the beta cells. Furthermore, it was noticed that the extracts had relatively the same onset of action as the standard drug glimepiride, which further supports the postulated mechanism.

Although the active metabolites in *Terfezia claveryi* were not fully identified in the present work, these compounds cannot be excluded from being involved in the antidiabetic activity of the tested crude extracts. They can react individually or in synergy to reduce hyperglycemia. The two major classes of compounds identified in truffle species, flavonoids and phenolic acids, are well-known for their insulin potentiation activity and enhancement of blood glucose uptake in peripheral tissues through GLUT-4 [33,34,35]. For example, apigenin controls hyperglycemia by raising the blood insulin levels, while rutin increases glucose uptake by peripheral tissues and improves insulin resistance [33,34]. Moreover, gallic acid induces glucose uptake peripherally by promoting GLUT-4 translocation [36]. Psicopyranose was also reported to alleviate postprandial hyperglycemia [37].

Polysaccharides in mushrooms have been considered prominent bioactive residents. In addition, the truffle is rich in low glycemic index sugars that were reported to have an antidiabetic effect. Treholase is a major sugar isolated from *Terfezia claveryi* with potential antidiabetic activity. It can improve insulin sensitivity by affecting the glucose signaling pathways or diminishing oxidative stress [38]. Mannitol is another sugar alcohol with reported glucose uptake activity. The in silico docking analysis suggested that the GLUT-4 transporter is a possible molecular target of mannitol [39]. However, several hypoglycemic mechanisms were identified, including reducing gut glucose absorption, enhancement of β-cell mass and potentiating insulin signaling [40]. 

In the current study, several phenolic compounds were detected in *Terfezia claveryi* extracts using UPLC-ESI-MS. Previous studies identified the presence of benzoic acid derivatives (*p*-hydroxybenzoic, vanillic, syringic, gentisic and protocatechuic acids) and cinnamic acid derivatives (*p*-coumaric, ferulic and p-hydroxycinnamic acids) were detected, in addition to apigenin and rutin flavonoid derivatives. All were reported to possess α-glucosidase inhibitory activity [33,35,40].

It has been reported that the unsaturated linoleic acid and α-linolenic acid, as well as the saturated palmitic acid detected in the *T. claveryi* macerate, and the infusion in addition to the stearic acid detected in all the extracts was identified by a multivariate model as α-glucosidase inhibitors, later verified by an in vitro enzymatic assay [41]. The reported IC_50_ for palmitic and stearic acid were (0.749 and 1.23 mM), respectively [42]. Furthermore, the same effect was reported for trans-vaccenic acid, an unsaturated fatty acid identified in *Terfezia claveryi* decoction and the microwave-assisted extract. The other saturated fatty acids identified in the extracts had higher IC_50_ values, as reported in the literature [42].

Moreover, the *T. claveryi* is a precious source of unique proteins and peptides unrevealed in other animals, plants and microorganism sources. Thus, they were reported to have multiple beneficial effects, including antidiabetic activities [43]. Therefore, future studies are encouraged to investigate the different polysaccharides and peptides found in *Terfezia claveryi.*

It was noticeable that the higher dose of the extracts (500 mg/kg body weight) did not significantly reduce the blood glucose level compared to the lower dose of 250 mg/kg body weight. The above-observed effect was reported before. The extract may have been up taken to the system through a saturable transport, where at a specific concentration saturation occurs, and the rest is excreted [44]. In addition, the solubility at higher doses may be affected, causing a decrease in drug absorption to the systemic circulation. Thus, the antihyperglycemic effect may be attained at doses lower than 500 mg/kg. 

The antihyperglycemic effect of the microwave-assisted extract and decoction extended beyond four hours (single dose of 250 mg/kg body weight). On the other hand, the sugar level increased in animal groups treated with the macerate and the infusion. 

Previous investigation of the methanolic extract of *Terfezia claveryi* slowed down glucose absorption by inhibiting the α-amylase enzyme. The extract effect was assessed in diabetic rats where the fasting blood glucose level was reduced compared to the control [16]. The ethanolic extract of *Terfezia boudieri* reduced blood glucose levels significantly in diabetic rats with a higher survival rate in the treated animals [45]

In this study, the administered aqueous crude extracts of *Terfezia claveryi* exhibited antihyperglycemic activity. However, it is worth identifying the major constituent(s) responsible for the activity and investigating the detailed hypoglycemic mechanism(s).

## 4. Materials and Methods

### 4.1. Materials and Reagents

Alpha-glucosidase (CAS: 9001-42-7), alloxan (CAS: 2244-11-3) and all other chemicals and reagents used in this study were of analytical grade supplied by Sigma-Aldrich. Glimepiride was purchased from a local pharmaceutical company.

### 4.2. Plant Material

The tubers of *Terfezia claveryi* were bought from the local market in Amman city in (January 2019). The botanical identification and authentication of the plant were performed by Prof. Mayadah Shehadeh, School of Pharmacy, University of Jordan. The voucher specimen with the number (TCMSAA 2018) was deposited in the University herbarium for future reference. 

### 4.3. Preparation of Crude Plant Extract

The plant’s tubers were washed with distilled water, cleaned and peeled. Then, it was grounded using an electric mixer grinding machine. The grounded tubers were macerated in distilled water for 2 h with continuous shaking at room temperature. The other portions were decocted for 15 min, microwaved for 8 min (900 W) or infused with continuous shaking for 2 h at room temperature. The aqueous crude extract was obtained by filtration (using Whatman filter paper, 125 mm). The crude extracts were lyophilized at 80 °C (using the Lyophilizer, SP Scientific, USA) to yield 154 gm (macerate), 2.0 gm (infusion), 3.0 gm (microwave-assisted), and 3.0 gm (decoction). The lyophilized extracts were stored at 4 °C in the dark until needed. 

### 4.4. Microscopical Characterization

One gram of the dried *Terfezia claveryi* powder was mounted in chloral hydrate, according to [17]. Photomicrographs were taken using a light microscope with a built-in camera (Leica DM750).

### 4.5. Qualitative Phytochemical Screening

The macerated extract was subjected to qualitative phytochemical screening for the presence of some chemical constituents. Phytochemical tests were carried out by adopting standard procedures [46].

### 4.6. Determination of Total Phenolic Content of the Extracts

The total phenolic content of the aqueous extracts was determined with the Folin–Ciocalteu colorimetric method adapted from [47], with minor modifications. Briefly, 26 µL of the extract solution was mixed with 13 µL of 10% (*w/v*) Folin–Ciocalteu reagent. After 5 min, 67 µL of Na_2_CO_3_ (8%) and 70 µL of distilled water were subsequently added to the mixture and incubated at room temperature for 30 min in the dark with agitation. Afterward, the absorbance was measured at 765 nm. The total phenols content was determined using a calibration curve prepared with gallic acid standard as a reference. The values were reported as mg of gallic acid equivalent (GAE) per gram dry weight of the extract (mg GAE/g). The mean values were obtained from triplicate experiments.

### 4.7. Determination of α-Glucosidase Inhibition Activity

The α-glucosidase inhibitory activity of the aqueous extracts was performed according to a previous study [48] and adapted in a 96-well plate. Briefly, 50 µL of 100 mM sodium phosphate buffer (pH 6.98), 50 µL of varying concentrations of extract, 30 µL of enzyme solution (α-glucosidase 2 IU/mL) and Saccharomyces cerevisiae (Sigma-Aldrich, USA) were added. The mixture was incubated for another 30 min at (37 °C). Next, the substrate (p-nitrophenyl-β-D-glucopyranoside,10 mM) was added, mixed and incubated at 37 °C for 5 min. Finally, 50 µL of pH 12 sodium phosphate buffer was added to stop the reaction. The absorbance of 4-nitrophenol formed during the reaction was registered at a wavelength of 410 nm. The blank with 100% enzyme activity was prepared by replacing the extract with the buffer. A blank reaction was similarly prepared using the plant extract at each concentration in the absence of the enzyme solution. A positive control sample was prepared using acarbose (Bayer), and the reaction was performed similarly to the reaction with plant extract, as mentioned above.

The inhibition of α-glucosidase was expressed as a percentage of inhibitory and was calculated by the following formula:

$$\%\;\mathrm{Enzym}\;\mathrm{inhibition}=\frac{\left(\mathrm{Abs}\;\mathrm{control}-\mathrm{Abs}\;\mathrm{test}\right)}{\mathrm{Abs}\;\mathrm{control}}\times 100$$

The % α-glucosidase was plotted against the extract concentration and the IC_50_ values were obtained from the graph. The mean values were obtained from triplicate experiments.

### 4.8. UPLC-ESI-MS

An amount of 10 mg of each dried plant extract was weighed and then solubilized in 100 µL DMSO (dimethyl sulfoxide). The extracts were diluted up with methanol: water (80:10 %), vortexed and centrifuged at 3000 rpm for 10 min. Finally, the samples were injected into the system. All solvents used were of HPLC grade.

The test samples were analyzed by the Bruker impact II ESI-Q-TOF system, provided with Bruker Dalotonik Elute UPLC system and characterized by mass accuracy of less than one ppm; the mass resolution was 50,000 FSR (Full Sensitivity Resolution) and the TOF repetition rate was up to 20 kHz.

Chromatographic separation was run through the Bruker Solo C 18-2 column with a size of 100 mm × 2.1 mm and with a particle size of 2 μm. The mobile phase comprises water, formic acid (HCOOH, 0.05%) and acetonitrile. The linear gradient started with water–formic acid (5–80%) from 0 to 27 min. Then, 95% acetonitrile (27–29 min) and 5% acetonitrile (29.1 min). The analysis was carried out using MetaboScape version 5.

### 4.9. Experimental Animals

Forty healthy adult (6–10 weeks of age) male BALB/c mice weighing between (18–23 g) were purchased from the Animal House of the Applied Science Private University. Animal care and use were conducted according to standard ethical guidelines, and all experimental protocols were approved by the Research and Ethical Committee at the University of Jordan (approval number: 19/2021/205).

The mice were maintained under standard conditions. The animals were kept in suitable cages and maintained under (12 h of light and 12 h of the dark cycle, 22–25 °C, 45–65% humidity) following the in-house ethical guidelines for animal protection. They were fed a high-fat diet (consisting of 30% animal fat, 10% glucose and 60% basic diet (*w/w*)) and were allowed free access to water ad libitum. After a randomized grouping and before the initiation of the experiment, the animals were acclimatized to the laboratory conditions for seven days.

### 4.10. Induction of Experimental Diabetes

Diabetes was evoked 45 days after a high-fat diet. Alloxan was dissolved in distilled water at a dose of (130 mg/kg) and intraperitoneally administered to overnight fasted (16 hrs) mice every week for three consecutive weeks. Thirty minutes later, food and water were allowed freely to the animal. A 5% glucose solution was given to the animal to prevent death from hypoglycemic shock.

The diabetic condition was confirmed after 72 hr to one week of injections by noticing the symptoms of polyuria and polydipsia. Animals that showed permanent high fasting blood glucose levels (FBGL) above 130 mg/dl were considered to be diabetic and were included in the study

### 4.11. Grouping and Dosing of Animals

After the experimental mice became diabetic, they were assigned randomly into six groups. Each group contained six mice (*n* = 6).

Group I: diabetic mice were given intraperitoneal normal saline (0.3 mL).

Group II: diabetic mice were given intraperitoneal glimepiride (1 mg/kg).

Group III: diabetic mice were given extract obtained by maceration (500 mg/kg).

Group IV: diabetic mice were given extract obtained by decoction (500 mg/kg).

Group V: diabetic mice were given extract obtained by infusion (500 mg/kg).

Group VI: diabetic mice were given extract obtained by microwave-assisted extract (500 mg/kg).

After one week the experiment was repeated using a lower dose (250 mg/kg).

All extracts were dissolved in normal saline and directly administered intraperitoneally.

Blood sugar level was measured for each animal just before treatment (at 0 min) as a baseline measurement and then at 30, 60, 90,120, 180, 240 and 360 min post-treatment. Free access to water and food was allowed after intraperitoneal administration of the extracts and glimepiride. Glimepiride was selected as a standard drug for the study based on earlier studies.

The blood glucose level was measured from cut tail tips and was determined using the glucometer (Accu-Check^®^ Performa, Germany). The percentage change of blood glucose level from the initial glycemia was calculated using the following formula:

$$\%\;\mathrm{Glycemic}\;\mathrm{change}\;=\frac{\left(\mathrm{Gx}\;-\;\mathrm{Go}\right)}{\mathrm{Go}}\times 100$$

where G_o_ is the initial blood glucose value at zero time after six hours of fasting (mg/dL) and G_x_ is the blood glucose value at x minutes after vehicle or tested compounds administration (mg/dL) [49].

### 4.12. Statistical Analysis

In vivo biological evaluation results were expressed as the means ± standard error of means (SEM) for all 6 mice in each group. Statistical differences between the treated and the control groups were performed using the Statistical Package for Social Science (SPSS) version 24 software. Between-group and within-group analyses were carried out using one-way analysis of variance (ANOVA), followed by Tukey’s Kremer multiple comparison tests. A difference in the mean values of (*p* = 0.05) or less was considered statistically significant.

## 5. Conclusions

To best of our knowledge, this study is the first to determine the microscopical fingerprinting and to investigate the effect of *Terfezia calveryi* aqueous crude extracts prepared using different extraction methods (maceration, infusion, decoction and microwave-assisted extract technique). The crude extracts have shown a significant glucose-lowering activity in the high-fat diet alloxan-induced diabetic mice. The potential antidiabetic activity of the truffle extracts may be related to the decrease in the absorption of carbohydrates from food, as suggested by the α-glucosidase inhibitory activity, and it could be due to the direct stimulation of insulin secretion from pancreatic β-cells.

Therefore, this study supports the use of *Terfezia claveryi* as part of the management of hyperglycemia in patients with diabetes mellitus type 2. However, further studies are required to identify the lead bioactive compound(s) responsible for the antidiabetic activity, along with its molecular mechanism, in the pathophysiology of diabetes.

## Figures and Tables

**Table 1:** Detected compounds in different *Terfezia claveryi* aqueous extracts using UPLC-ESI-MS.

No.	Compound	Molecular Formula	*m/z*	Retention Time	Extract Chemical Composition			
					Maceration	Decoction	Infusion	Microwave-Assisted
1	Isoferulic acid	C_10_H_10_O_4_	193.051	1.22	ND	ND	ND	+
2	Succinic acid	C_4_H_6_O_4_	117.01981	1.68	+	ND	ND	ND
3	Protocatechuic aldehyde	C_7_H_6_O_3_	137.02479	2.09	+	ND	ND	+
4	Coumaric acid	C_9_H_8_O_3_	163.04002	2.53	+	ND	ND	ND
5	Vanillic acid	C_8_H_8_O_4_	167.03451	3.09	+	+	ND	+
6	Caffeic acid	C_9_H_8_O_4_	179.03501	3.19	ND	ND	ND	+
7	Scopoletin	C_10_H_8_O_4_	191.03477	4.29	+	ND	ND	ND
8	Palmitic acid	C_16_H_32_O_2_	255.23259	30.23	+	ND	+	ND
9	Trans-vaccenic acid	C_18_H_34_O_2_	281.24855	30.34	+	ND	+	ND
10	Stearic acid	C_18_H_36_O_2_	284.27123	33.18	+	+	+	+

ND: not detected, +: detected.

**Table 2:** α-glucosidase inhibition activity and IC_50_ by different aqueous extracts of *T. claveryi* and acarbose.

Method of Extraction	IC_50_ (mg/mL)	% Inhibition
Maceration	3.31 ± 5.83	75.9 ± 3.66
Decoction	5.18 ± 1.04	58.4 ± 1.35
Microwave-assisted	2.43 ± 0.02	100 ± 0.02
Infusion	3.26 ± 0.99	81.5 ± 1.22
Acarbose (Control)	0.33	75.0

Values are expressed as mean ± standard deviation.

**Table 3:** Percentage change of blood glucose after a single dose administration of *Terfezia claveryi* crude extracts.

Dose	Treated Group	% Change in Blood Glucose Level after (minutes) of Treatments						
		30	60	90	120	180	240	360
250 mg/kg	Macerate	8.1 ± 0.12	37.4 ± 0.21	22.3 ± 0.13	4.1 ± 0.07	−21.3 ± 0.05 *	−29.4 ± 0.05 *	−26.6 ± 0.04 *
	Decoction	13.8 ± 0.07	33.9 ± 0.17	25.5 ± 0.21	9.5 ± 0.15	1.1 ± 0.13	−13.2 ± 0.07 *	−8.7 ± 0.08 *
	Microwave-assisted	37.5 ± 0.13	57.9 ± 0.23	37.6 ± 0.17	17.2 ± 0.10	−1.9 ± 0.10	−12.6 ± 0.06 *	−17.3 ± 0.04 *
	Infusion	44.5 ± 0.12	66.1 ± 0.11	35.7 ± 0.07	7.8 ± 0.06	−16.8 ± 0.09 *	−24.8 ± 0.06 *	−27.8 ± 0.05 *
500 mg/kg	Macerate	4.6 ± 0.10	71.8 ± 0.08	61.0 ± 0.14	36.8 ± 0.09	−8.2 ± 0.09 *	−18.8 ± 0.11 *	−29.3 ± 0.05 *
	Decoction	39.0 ± 0.07	54.1 ± 0.07	35.8 ± 0.13	35.8 ± 0.07	3.8 ± 0.04	−0.24 ± 0.12	−2.7 ± 0.08 *
	Microwave-assisted	34.0 ± 0.08	61.4 ± 0.17	42.3 ± 0.16	33.0 ± 0.27	11.2 ± 0.12	−7.6 ± 0.07 *	−6.0 ± 0.07 *
	Infusion	33.0 ± 0.13	69.1 ± 0.15	56.0 ± 0.14	45.7 ± 0.20	−26.4 ± 0.03 *	−31.8 ± 0.05 *	−32.4 ± 0.08 *
	Negative control	23.1 ± 0.11	24.9 ± 0.07	39.1 ± 0.18	42.2 ± 0.17	32.7 ± 0.10	33.8 ± 0.08	32.5 ± 0.13
	Glimepiride	35.0 ± 0.07	29.6 ± 0.07	5.8 ± 0.09	−2.8 ± 0.05	−8.4 ± 0.05	−18.1 ± 0.05	−25.1 ± 0.05

* The significance level *p* < 0.05 versus negative control: normal saline; FBG: fasting blood glucose (mg/dl). There was no significant difference between the extract at both doses versus glimepiride.

## Data Availability

Not applicable.

## Sample Availability

Samples of the extracts are available from the correspondence.
